# The Effects of a Parent-Focused Social Media Intervention on Child Sun Safety: Pilot and Feasibility Study

**DOI:** 10.2196/48402

**Published:** 2023-12-08

**Authors:** Sharon Manne, Yelena Wu, David Buller, Carolyn Heckman, Katie Devine, Sara Frederick, Justin Solleder, Alexis Schaefer, Shou-En Lu

**Affiliations:** 1 Behavioral Sciences Rutgers Cancer Institute of New Jersey Rutgers, The State University of New Jersey New Brunswick, NJ United States; 2 Huntsman Cancer Institute University of Utah Salt Lake City, UT United States; 3 Klein Buendel, Inc Lakewood, CO United States; 4 Rutgers School of Public Health New Brunswick, NJ United States

**Keywords:** health behavior, health promotion and prevention, parenting, prevention science, parents, parent, Facebook, social media, sun, prevention, skin, dermatology

## Abstract

**Background:**

Middle childhood (ages 8-12 years) is a critical period for forming behavioral habits and reducing the risk for the development of skin cancer later in life. During this time, children develop more autonomy and spend more unsupervised time away from their parents. Professional agencies recommend that all children engage in regular sun protection behaviors and avoid the sun during peak daytime hours. Unfortunately, in middle childhood, child sun protection often declines and UV radiation exposure increases. Effective parenting involves balancing ways to encourage the child’s increasing independence while providing practical assistance to ensure sun protection is implemented.

**Objective:**

The goal was to evaluate the feasibility, acceptability, and preliminary effects of Sun Safe Families, a Facebook group intervention for parents of children between 8 and 12 years of age.

**Methods:**

The team developed Facebook messages targeting parent knowledge, normative influences, sun safety barriers, planning and goal setting, confidence in implementing sun safety, communication, forming habits, and managing sun safety in risky situations. A total of 92 parents were enrolled, and the groups ran for 6 weeks. Feasibility was measured by enrollment and retention rates. Acceptability was measured by engagement in the Facebook groups. Satisfaction was assessed by a treatment evaluation. At pre- and post-intervention, parents completed measures of child sun protection, UV radiation exposure, sunburn, sun safety knowledge, child risk, barriers, sun protection self-efficacy, planning, sun safe habits, norms for child sun safety, and communication about sun safety.

**Results:**

Enrollment (64.3%, 92/143) and retention (94.6%, 87/92) were good. On average, participants viewed 67.6% (56.8/84) of posts, “liked” 16.4% (13.77/84) of posts, commented on 14.8% (12.43/84) of posts, and voted on 46% (6.4/14) of polls. Satisfaction was excellent. From pre- to post-intervention, there were significant increases in child sun protection, sun exposure, and sunburn (*P*<.01; moderate effect sizes), as well as statistically significant increases in planning and self-efficacy (*P*<.05) and family norms and parent communication (*P*<.01).

**Conclusions:**

This study demonstrated high survey retention, acceptability, and satisfaction with the intervention. There were promising preliminary effects on child sun protection behaviors and parent sun protection attitudes and communication with their child. Replication with a larger sample size and a comparison condition is warranted.

## Introduction

Childhood is a critical period for forming sun safety habits and reducing the risk of the development of skin cancer later in life. Approximately 25% of total lifetime sun exposure occurs before the age of 21 years, and sunburns in childhood are frequent [1,2]. Thus, skin is at increased susceptibility to carcinogenic effects of UV radiation during childhood and adolescence. Exposure to UV radiation in sunlight is the only modifiable risk factor for both melanoma and nonmelanoma skin cancers [3]. Sun safety is recommended for children by all cancer-focused professional agencies [4-7]. The US Preventive Services Task Force recommends counseling children and their parents about minimizing exposure to UV radiation for persons aged 6 months to 24 years with fair skin types, to reduce their risk for skin cancer [5,7].

Given the elevated risk, it is unfortunate that many children do not engage in recommended sun protection. Use of sunscreen (9%-77%); wearing a long-sleeved shirt (5%-40%), long pants (18%-35%), a hat (5%-64%), or sunglasses (3%-64%); seeking shade (38%-83%); and avoidance of the sun (23.6%-51%) are less than ideal [8]. Up to 80% of children have sunburns over the course of a single summer, increasing the likelihood that they will develop skin cancer later in life [8-11].

Sun protection and UV radiation exposure are significant problems during middle childhood, defined as 8 to 12 years of age and often referred to as the “tween” years. During this developmental period, the use of sun protection behaviors declines, and UV radiation exposure and sunburn occurrence increase [10,12-15]. As children approach adolescence, they develop more autonomy and spend more unsupervised time away from their parents [12,16-18]. While parents continue to carry responsibility for the child’s sun protection, child participation in the management of their health behaviors during the preteen years increases. Child sun safety becomes a negotiated process for parent and child and thus, more challenging for parents [16,17].

Despite the importance of establishing optimal sun safety habits for children during middle childhood, there are few effective parent-focused interventions for them. Most prior interventions have occurred in schools and other community organizations and do not target parents [19-22]. There is a limited literature evaluating parent-focused interventions that include children in this age group, but no studies target the 8- to 12-year-old age group specifically. Although existing research has illustrated positive effects, this work has limitations. First, interventions have not targeted known determinants of child sun safety, such as building parent confidence and skills in managing the child’s sun safety. Second, prior work has utilized in-person counseling and printed education materials [20] or mailed educational information [19,20]. Reliance on in-person or mailed delivery limits the ability to disseminate the intervention on a broader scale, does not take advantage of the latest online communication technologies, and may include insufficient intervention dose to initiate and maintain behavior change. Third, their individual-level focus does not harness interpersonal influences such as support from other parents or foster effective communication with the child about sun safety. Finally, there are no studies specifically focusing on parents of 8- to 12-year-old children.

This study describes the development of the Sun Safe Families Facebook (SSF) intervention and a pilot study of its feasibility, acceptability, satisfaction, and impact. SSF focused on potentially modifiable parent factors associated with increased use of sun protection among children. SSF was delivered via social media. We chose social media as our delivery platform for parents over other technology-based approaches (eg, text and mobile intervention) because social media offers the opportunity to both give and receive input from parents, possibly enhancing the credibility of the advice [23,24]. We chose Facebook over other social media (eg, Instagram and YouTube) for 3 reasons. First, it is widely used by parents: 79% of US parents use Facebook [23-25]. Second, Facebook offers a private group option with an identified group moderator. A moderated group allowed a large number of parents of similar-age children to share experiences and provide support to one another while having a trusted source (group moderator) promoting factual information and evidence-based parenting strategies. Third, intervention content within a private group on Facebook can span several months to increase the intervention dose.

The primary aim of this study was to describe SSF’s feasibility, acceptability, and satisfaction. Feasibility was measured by enrollment and retention rates, acceptability was measured by engagement in the Facebook groups, and satisfaction was measured by treatment evaluation. The secondary aim was to assess the preliminary effects of the SSF intervention on primary (ie, parent-rated child sun protection, UV radiation exposure, and sunburn) and secondary individual and interpersonal factors. Individual factors included knowledge about child sun safety, perceived child skin cancer risk, barriers to child sun protection, sun protection self-efficacy, planning, and sun safe habits. Interpersonal factors included parent-rated peer and family norms for child sun safety and parent communication about sun safety with the child.

## Methods

### Sun Safe Families Facebook Message Content Development

Intervention content was guided by the Integrative Behavioral Model (IBM) [26,27] and Ecological Systems Theory (EST) [28,29], which propose that engagement in child sun protection is influenced by multilevel factors on the individual, interpersonal, and environmental levels. Individual-level factors included parent sun safety knowledge and attitudes. Interpersonal-level factors included peer and family norms and parent facilitation for and communication about sun protection. Environmental-level factors included settings where the child encounters UV radiation exposure and where sun safety is implemented. Intervention messages included information (eg, recommended child sun protection and links to reliable resources) and interactive content (eg, discussion questions and polls) to target key constructs in these models.

Key goals were to (1) improve parent knowledge about UV radiation exposure and child sun safety, (2) increase awareness of childhood as a critical period for prevention of skin cancer and child phenotypic or behavioral risks, (3) foster development of stronger family and peer normative influences for child sun safety by engaging group members in discussion, (4) reduce perceived sun safety barriers by focusing on ways to address barriers, (5) motivate planning and goal setting for child sun safety, (6) build confidence in the ability to implement child sun safety, (7) assist parents in implementing effective communication and facilitation practices to improve child sun safety, (8) assist parents in forming stronger sun safety habits, and (9) help parents manage child sun safety in risky situations. Messages also addressed risky situations such as beach days and other water activities. Additional interpersonal influences were targeted by fostering interaction between participants to foster group support for child sun safety.

**Figure 1:**
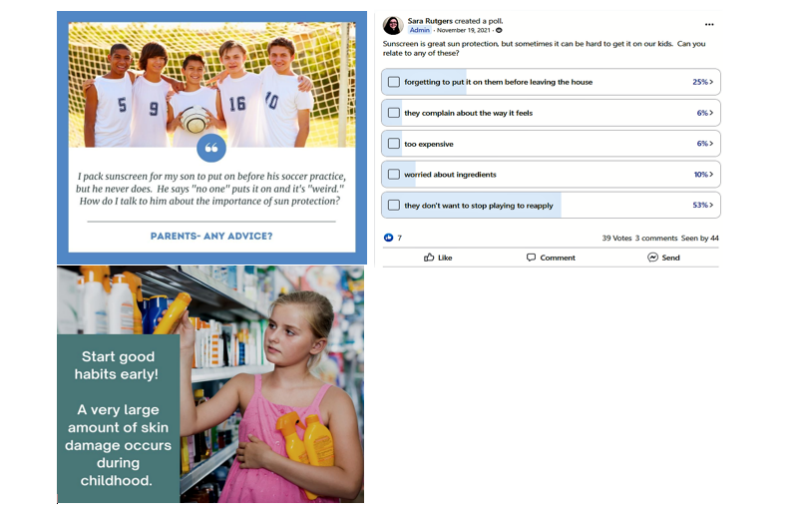
Samples of posts from the Sun Safe Families Facebook groups.

Our prior research using Facebook-delivered interventions illustrated the importance of messaging on non–sun-related topics to maintain engagement for long-term interventions [30]. Based on this work, about 17% (14/84) of the posts focused on topics relevant to parents, including child mental health, general health practices (eg, exercise), social concerns, and general parenting concerns. For this pilot study, we developed 84 messages to be delivered over the course of the 6-week intervention period. Topics are shown in Table 1 and sample posts are shown in Figure 1. The messages were posted in 2 Facebook groups—45 parents in group 1, which ran in the summer of 2021, and 47 parents in group 2, which ran in the early fall of 2021.

**Table 1:** Sun Safe Families intervention constructs, corresponding content, and the number of messages in the Facebook groups containing them.

Construct	Content	Number of messages (81-84 in total), n
Sun safety knowledge	Phenotypic risk for child Child risk from sunburns Child sun safety recommendations Child sun protection behaviors UV radiation exposure during daytime hours	20-22
Attitudes	Perceived risk for child developing skin cancer Barriers to implementing child sun protection Benefits for implementing child sun protection	2-3
Self-efficacy	Skills to manage child sun safety How to apply sunscreen	7
Planning	Planning sun safe outdoor activities for your family Planning for high UV radiation exposure settings	3-4
Habits	How to make sun safety a regular practice for your child	2-3
Family norms	Parent as a role model for the child Importance of sun safety as a family practice Advocating for sun safety in other settings	6-8
Peer norms	Friends and other parents’ sun safety Within group support for sun safety	3-4
Parent communication and facilitation	Handling child resistance and motivation Talking to a preteen about sun protection Setting up a sun safe environment Communicating with other family members about sun safety	3-4
High-risk settings	High UV radiation risk settings and situations	3
Non-sun safety	Mental health concerns and talking to the child about these topics Talking to child about tobacco use Body image concerns and how to support the child Managing children’s time online and engagement in social media Healthy eating habits for children, family dinner times, and food allergies Holiday memes	28-29

### Eligibility

Parents were considered eligible if they met the following criteria: (1) parent or guardian of a child aged 8-12 years, (2) parent-reported child sunburn in the last year or suboptimal sun protection on the Sun Protection Habits Survey [31] (<3 out of 5), (3) parent and child had no personal history of melanoma, (4) parent had a Facebook account and home internet, and (5) parent or guardian was able to read English.

### Recruitment, Consent, Enrollment, and Follow-Up Survey Procedures

Participants were recruited from a third party, Qualtrics (Qualtrics International Inc). Qualtrics uses an opt-in panel of respondents who have agreed to participate in research studies and partners with numerous online sample providers to supply a larger network of diverse, quality respondents. Thus, the majority of their respondents come from traditional, actively managed, and double-opt-in market research panels. Although this is their preferred method, social media is occasionally also used to gather respondents. To exclude duplication and ensure validity, Qualtrics checks IP addresses and uses unique, sophisticated digital fingerprinting technology. Respondents are invited to studies in various ways. For instance, potential respondents are sent an email invitation informing them that the study is for research purposes only, how long it is expected to take, and the amount of incentives. Other times, respondents will view the surveys they are likely to qualify for upon signing into a panel portal. Other common invitation methods include in-app notifications and SMS text message notifications. To avoid self-selection bias, invitations do not include specific details about the topic of the study and are very general. For this study, Qualtrics panel members received the link to the eligibility screening survey. Once the prospective participant’s screening eligibility was confirmed, contact information was passed to the study team who confirmed eligibility, explained the study procedures, and enrolled participants. Study staff then emailed a link to the consent and baseline survey to all eligible participants. Participants were informed of the study requirements, potential risks and benefits of study participation, a breakdown of study compensation via the study description screen in the survey, and a consent form. After completing the baseline survey, participants were assigned a study ID number and sent a request to join a private Facebook group. After the Facebook groups’ completion, participants were sent a link for the follow-up survey and intervention evaluation, which was completed online.

### Ethical Considerations

This study was reviewed and approved by the Rutgers University institutional review board (#2021000177). As stated above, participants underwent an informed consent procedure prior to completing the baseline survey. Participant data were deidentified in all data sets through the assigning of unique participant ID numbers. Due to the use of social media or Facebook, if a participant joined the private Facebook group, other participants could see any publicly available information on their Facebook account such as username or any information they disclosed in a post to the group. Participants were made aware of the risks of this prior to consenting, and the Facebook groups were made private so nonparticipant users could not see the participants’ posts within the group. Participants were compensated US $25 for completing the baseline survey and US $25 for the follow-up survey in the form of gift cards.

### Facebook Group Procedures

The SSF intervention was delivered to participants in 2 private Facebook groups of 45-47 parents each over 6 weeks. Posts were made twice a day, which is the frequency suggested by social media marketers to engage participants without overburdening them [32]. Participants’ membership and activities in the private Facebook group were only viewable by other invited group members and study staff. They were not publicly viewable to users outside the group.

Each group was moderated by a member of the study team. The moderator logged into the group 2 to 3 times a day to review activity and facilitate engagement by “liking” participant comments and replying with supportive responses. The moderator notified the study lead investigator if misinformed, distressing, or inappropriate comments were posted in the Facebook group. Because Facebook norms were for brief and frequent interactions, moderator responses were brief and were intended to engage participants in discussions, reinforce engagement (eg, using a “like” reaction), and answer questions. To ensure the privacy and safety of participants in the Facebook groups, the study team took the following measures. First, the study’s informed consent contained a description of the privacy settings for Facebook groups. Second, during the telephone contact, the research staff described what a “private” Facebook group meant in terms of privacy (eg, the group and its contents are not visible to the public), the privacy limitations for a “private” Facebook group (eg, cannot guarantee that other participants would not share content posted with others), and the confidentiality rules of the group. Third, if the study team learned that a participant had breached confidentiality, it was considered a study adverse event and the study team reviewed it and contacted the participant to discuss confidentiality. There were no breaches of confidentiality during the groups.

### Measures for the Primary Aim: Feasibility, Acceptability, and Satisfaction

Feasibility was measured by enrollment and retention rates. Because initial enrollment was from a third party (Qualtrics), we defined enrollment as the percentage of prospective participants provided to the study staff by Qualtrics who subsequently enrolled in the study. Acceptability was measured by engagement in the Facebook groups, which was assessed by the average percentage of posts viewed, the average percentage of posts liked, the average number of posts commented on, and the average percentage of polls completed. Satisfaction was assessed by an evaluation survey and open-ended questions.

The intervention satisfaction measure included 18 items asking parents to rate the helpfulness, value, relevance, and accuracy of the materials posted, as well as aspects of group participation such as comfort with participation, feeling connected with, actively involved with, enjoying expressing opinions about, and reading posts (1=not at all and 7=extremely). Sample items included “The information I received from the posts and group discussion was valuable,” “The information I received made it easier to talk to my child about engaging in better sun protection,” and “I could identify with other people in the group.” Additionally, 5 open-ended, free-text questions asked about (1) overall impressions of the group; (2) thoughts about topics; (3) thoughts about posts; (4) main reasons they did not participate, if they did not; and (5) the overall group.

### Measures for the Secondary Aim

#### Child and Parent Demographics and Child Phenotypic Risk

Parents reported their own and the child’s biological sex, date of birth, and race and ethnicity. Parents reported the child’s skin color (very fair, fair and olive, light brown, dark brown, or very dark) and tanning ability (“What would happen if this child’s skin was exposed to bright sunlight in the summer without any sun protection?”; response options: get very brown, get moderately tanned, get mildly or occasionally tanned, or get no suntan at all or only get freckled).

#### Child Sun Protection Behaviors

Using the Sun Habits Survey [31] parents rated how often the child engaged in 6 behaviors between 10 AM and 4 PM in the last 3 months: wore sunscreen with sun protection factor (SPF) >15, wore long pants, wore a long-sleeved shirt, wore sunglasses, wore a wide brim hat, and reapplied sunscreen every 2 hours if outside or in the water (1=never and 5*=*always). An average score was used in the analyses. Internal consistency was good at both time points: Cronbach α_pre-intervention_=.72 and Cronbach α_post-intervention_=.72.

#### Child Sun Exposure

Using the Sun Protection Habits Survey [31], parents reported how long the child was in the sun between 10 AM and 4 PM on a typical weekday in the last 3 months (1=30 min a day and 7=6 or more h per day). A separate item assessed exposure between 10 AM and 4 PM on a typical weekend in the last 3 months.

#### Child Sunburn

Parents reported how many times the child had a red and/or painful sunburn lasting a day or more in the last year (pretest) and 3 months (posttest), with the response options of 0 times, 1 time, 2 times, 3 times, 4 times, and 5 or more times [31].

#### Parent Knowledge About Child Sun Safety

A total of 10 true-or-false items created for this study assessed general knowledge about child sun safety (eg, “If you use a sunscreen SPF 30, your child can stay in the sun 30 times longer before receiving a sunburn than without sun protection” and “Protection in the sun is important for very young children [under 5 years]”). A total number of correct answers was calculated (score range 0-10).

#### Perceived Child Risk

The 2 items adapted from our prior work [30] asked parents to rate the child’s risk for skin cancer in the child’s lifetime and the child’s risk compared to others (1=very likely and 5=very likely). Internal consistency was excellent: Cronbach α_pre-intervention_=.90 and Cronbach α_post-intervention_=.88.

#### Perceived Barriers to Child Sun Protection

A total of 12 items adapted from prior work [33] assessed parent barriers to implementing sun protective behaviors with the child: forgetting to bring sun safety products; forgetting to remind the child to engage in sun protection; considering products too expensive, messy, and greasy; lack of assistance from partner and others; takes too much time; child resists, argues, or ignores parent; worry about chemicals in sunscreen; and feeling it is too hot to wear protective clothing (1=strongly disagree and 5=strongly agree). A mean was calculated, and higher scores indicated more barriers. Internal consistency was good: Cronbach α_pre-intervention_=.82 and Cronbach α_post-intervention_=.86.

#### Self-Efficacy for Child Sun Protection

A total of 8 items adapted from prior work [34] assessed parent confidence fostering child sun protection behaviors (eg, “Make sure they wear long pants or other clothing that reaches the ankles” and “Make sure they wear sunglasses”; 1=not at all confident and 5=very confident). A mean was calculated, and higher scores indicated more efficacy: Cronbach α_pre-intervention_=.85 and Cronbach α_post-intervention_=.87.

#### Planning for Sun Protection

A total of 3 items based on prior work [18,35] assessed the degree to which the parent made detailed plans about what they need to do to do a better job with the child’s sun protection, what behaviors to focus on, and how to talk to the child about sun safety (1=strongly disagree to 7=strongly agree) [18,35]. Higher scores indicated more planning. Internal consistency was excellent: Cronbach α_pre-intervention_=.92 and Cronbach α_post-intervention=_.93.

#### Child Sun Protection Habits

A total of 4 items were adapted from an existing scale [36] to assess the degree to which parent facilitation of their child’s sun protection behaviors is automatic or a habit (eg, “I start doing it before I realize I am doing it”) (1=strongly disagree to 5=strongly agree)*.* A mean was calculated, and higher scores indicated more automaticity. Internal consistency was excellent: Cronbach α_pre-intervention_=.91 and Cronbach α_post-intervention_=.92.

#### Peer Norms

A total of 8 items adapted from Rice and Klein [37] assessed the perceived attitudes of peers about sun protection (eg, “Other parents I know think that making sure my child uses sun protection is a good thing to do” and “Most people who are important to me think I should make sure my child uses protection”; 1=strongly disagree and 5=strongly agree)*.* A mean was calculated, and higher scores indicated more positive norms for sun protection. Internal consistency was good: Cronbach α_pre-intervention_=.85 and Cronbach α_post-intervention_=.86.

#### Family Norms

A total of 9 items based on our prior work [38] assessed perceived family attitudes toward sun safety (eg, “My family goes to all lengths to protect themselves from the sun” and “My family disapproves of people who tan.”; 1=strongly disagree and 5=strongly agree) [39]. A mean was calculated, and higher scores indicated more positive norms for sun protection. Internal consistency was satisfactory: Cronbach α_pre-intervention_=.68 and Cronbach α_post-intervention_=.68.

#### Facilitation and Communication

A total of 11 items developed specifically for this study assessed parent or child facilitation and communication about sun protection (eg, “Remind my child to use sun protection,” “Keep sun protection products and equipment handy when we are going outside in the sun,” and “My child and I come up with ways to make it easier for them to use sun protection”; 1=rarely and 5=always) [40,41]. A mean was calculated, and higher scores indicate more facilitation. Internal consistency was excellent: Cronbach α_pre-intervention_=.91 and Cronbach α_post-intervention_=.92.

### Approach to Analyses

Descriptive statistics (means, SDs, and frequencies) were used to characterize feasibility, acceptability, and satisfaction. Feasibility was defined as study enrollment and follow-up survey completion. The benchmark for enrollment was set at >60%, and the benchmark for follow-up survey completion was >80%. Acceptance was defined as participation in the Facebook groups. Since there are no standards for engagement in Facebook intervention, we did not set a benchmark. Satisfaction was defined as the average score on the intervention evaluation satisfaction measure, and we set a benchmark of an average of 6 on the 7-point Likert scale. Responses to the open-ended question about the group content or topics were categorized into themes and the number of comments in each theme was calculated.

Analyses for the secondary aim evaluated pre- and post-intervention differences using mixed model analysis. The participant was included in the statistical model as the random effect to account for the intra-person correlation between repeatedly measured outcomes. The means of the outcomes were estimated and compared using linear contrasts. A *P* value of .05 was set for significance testing.

## Results

### Sample Characteristics

Participant characteristics (92 parents and 92 children) are shown in Table 2. The majority of parents (73.9%, n=68) were female and about half (51.1%, n=47) of the children were female. About 80% of parents (n=78) and children (n=74) were White. Parents were relatively well-educated (56%, n=53 had completed a college or advanced degree) and married or cohabitating (70%, n=65). The majority of children (74%, n=68) had experienced a sunburn in the last year, 57% (n=52) had light or very light skin tone, and about a third (32%, n=29) had high sun sensitivity (ie, get mildly or occasionally tanned or get no suntan at all or only get freckled).

**Table 2:** Sample characteristics of participants who enrolled in the study and their children.

Characteristics			Parent (n=92)		Child (n=92)
Age (years), mean (SD)			40.3 (8.0)		11.0 (1.4)
Sex (female), n (%)			68 (73.9)		47 (51.1)
**Race, n (%)**					
	White	78 (84.7)		74 (80.4)	
	Black	6 (6.5)		8 (8.7)	
	Asian	5 (5.4)		4 (4.3)	
	Mixed	1 (1.1)		5 (5.4)	
	Other	1 (1.1)		1 (1.1)	
**Ethnicity, n (%)**					
	Hispanic or Latino	15 (16.3)		15 (16.3)	
	Non-Hispanic	77 (83.7)		77 (83.7)	
**Education, n (%)**					
	High school or lower	16 (17.4)		N/A^a^	
	Partial college	23 (25.0)		N/A	
	College degree	38 (41.3)		N/A	
	Graduate degree	15 (16.3)		N/A	
**Marital status, n (%)**					
	Married	55 (59.8)		N/A	
	Cohabitating	10 (10.9)		N/A	
	Divorced or separated	10 (10.9)		N/A	
	Single	15 (16.3)		N/A	
	Widowed	1 (1.1)		N/A	
	Missing	1 (1.1)		N/A	
**Skin tone, n (%)**					
	Very fair	N/A		11 (12.0)	
	Fair	N/A		41 (44.6)	
	Olive	N/A		20 (21.7)	
	Light brown	N/A		17 (18.5)	
	Dark brown	N/A		3 (3.3)	
**Sun sensitivity, n (%)**					
	Get very brown and deeply tanned	N/A		25 (27.2)	
	Get moderately tanned	N/A		37 (40.2)	
	Get mildly or occasionally tanned	N/A		25 (27.2)	
	Get no suntan at all or get freckled	N/A		4 (4.3)	
	Missing data	N/A		1 (1.1)	

^a^N/A: not applicable.

### Feasibility, Acceptability, and Satisfaction

#### Feasibility

**Figure 2:**
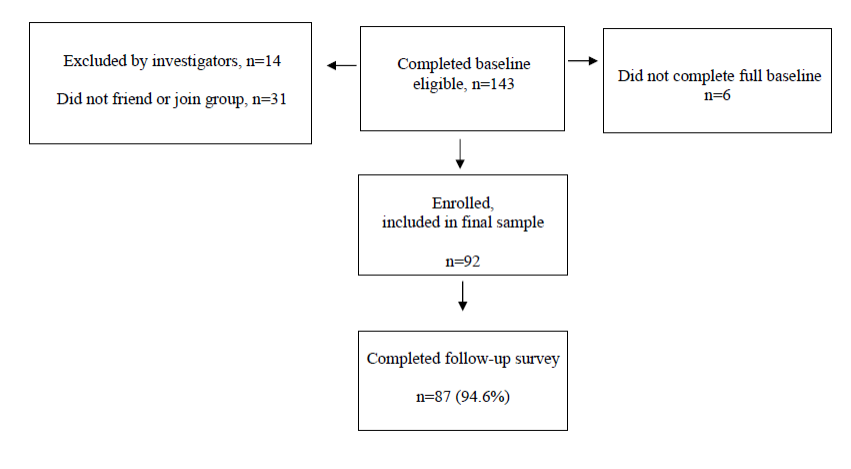
Sun Safe Families study CONSORT (Consolidated Standards Of Reporting Trials) flow diagram.

The Consolidated Standards of Reporting Trials (CONSORT) flow diagram is shown in Figure 2*.* A total of 143 eligible parents with children aged 8 to 12 years were identified from Qualtrics. Of these parents, 92 completed the survey, friended the team’s Facebook account, and joined the group. A total of 31 parents did not friend the team’s Facebook account or join the Facebook group. About 20 parents were excluded because they did not fully complete the baseline survey, did not provide the child’s date of birth, provided suspicious data, or had suspicious Facebook profile inconsistencies. The final enrollment rate, defined as prospective participants passed to the study team by Qualtrics who enrolled in the trial, was 64.3% (92/143). Comparisons between the 92 participants and 31 parents who were not included in the final sample on available baseline data did not illustrate any significant differences. In terms of retention, of the 92 parents in the sample, 87 (94.6%) completed the follow-up survey. Thus, the retention rate exceeded the benchmark of >80%.

#### Acceptability

The average number of posts viewed was 67.6% (SD 34.7%; 56.8/84), the median percentage of posts viewed was 85% (IQR 40%-89%) and 41% (38/92) of the sample viewed at least half of the posts. The average number of posts liked was 16.4% (SD 22.4%; 13.77/84), the median percentage of posts liked was 4% (IQR 0%-28.5%), and 10.8% (10/92) of the sample liked at least half of the posts. The average number of posts commented on was 14.8% (SD 19.4%; 12.43/84), the median percentage of posts commented on was 6% (IQR 11.9%-21.6%), and 7.6% (7/92) of the sample commented on at least half of the posts. The average number of polls voted on was 46.1% (SD 36.9%; 6.4/14), the median percentage of polls voted on was 43.6% (IQR 8.3%-83.3%), and 41% (38/92) of the sample voted at least half of the polls. At 67.6% (56.8/84), the percentage of posts viewed was higher than our benchmark of 50%. A summary variable named Facebook engagement was calculated by an average of the percentage of posts viewed, liked, commented on, and voted on (mean 36.2%, SD 24.2%).

#### Satisfaction

The treatment evaluation results are shown in Table 3. The average scale score was 6.19 (SD 0.87; 7=extremely helpful). Participants felt the messages were helpful (mean 6.3, SD 1.1; 7=extremely helpful), valuable (mean 6.4, SD 1.0; 7=strongly agree), contained accurate information (mean 6.5, SD 0.90; 7=strongly agree), and helped them feel more prepared to discuss sun protection with their child (mean 6.1, SD 1.1; 7=strongly agree). In terms of the group climate, participants felt comfortable participating (mean 6.2, SD 1.3), could identify with other group members (mean 5.2, SD 1.1), and enjoyed reading others’ comments (mean 6.4, SD 1.0). The average scale mean for the satisfaction measure exceeded the benchmark of 6. Overall impressions of the group described in open-ended questions were categorized into 6 categories: enhancing knowledge, sense of community, sharing content with family, communication with the child, addressing child resistance, and dislikes. Frequencies are shown in Table 4. There were 76 comments. The most common category of comments was knowledge (61.8%, n=47), followed by communicating with preteens (14.5%, n=11), fostering a sense of community (5.3%, n=4), sharing content with family (5.3%, n=4), and handling child resistance (2.6%, n=2). There were 6 negative comments (7.9%, n=6).

**Table 3:** Sun Safe Families Facebook intervention participant reported feedback^a^.

Feedback category			Score, mean (SD)
**Intervention**			
	Helpful	6.3 (1.1)	
	Learned something new	6.3 (1.1)	
	Information was interesting	6.3 (1.2)	
	Valuable	6.4 (1.0)	
	Easy to understand	6.6 (0.75)	
	Accurate	6.5 (0.90)	
	Relevant to child	6.5 (0.88)	
	Made easier to talk to child about sun safety	6.2 (1.0)	
	Helped understand how better sun protection may benefit child	6.4 (0.93)	
	Felt more prepared to talk to child about sun protection	6.1 (1.1)	
	Likely to improve child’s sun protection	6.1 (1.2)	
**Group experience**			
	Felt comfortable participating in discussion	6.2 (1.3)	
	Felt actively involved in group	5.6 (1.8)	
	Could identify with posts	6.1 (1.2)	
	Could identify with other group members	6.2 (1.2)	
	Enjoyed expressing opinions	5.7 (1.7)	
	Paid attention to other people’s comments	6.4 (1.0)	
	Enjoyed reading other people’s comments	6.4 (1.0)	
	Enjoyed reading posts	6.4 (1.0)	

^a^1=strongly disagree, 7=strongly agree.

**Table 4:** Sun Safe Families intervention specific feedback from participants.

Topic	Sample comments	Number of comments, n
Knowledge	I have a swimming pool in my backyard so me and my son is in the sun a lot and this group has taught me how important it is to use sunscreen and to reapply it. I learned a lot of new things. It was fun to interact and also learn what others were doing and what worked for them. Helpful in making me more aware of skin protection and ways to help prevent being in the sun and/or more ways to help protect my and my children’s skin when in the sun. My son is a lot lighter than me so this group has helped me to identify proper and better ways to keep my son and daughter protected from the sun.	47
Handing child resistance to sun safety	My son resists sunscreen and it’s difficult to get him to use it. He’s African American and I worry that he and his father (we are divorced) think he doesn’t need sun protection. Realized we were doing better than I thought, getting him to wear a hat was a struggle, but by sharing the group information with him I finally won him over.	2
Communicating with child	Appreciated when the topic was how to talk to your pre-teen about sun safety because it seems sometimes like little gets through. Being of woman of color, I notified my child that anyone can get sunburned. I loved the tips for talking to your kid about sun safety because, it opened up a whole new dialog for my kids and I	10
Sense of community	I enjoyed being able to talk to other parents about the struggles and challenges of getting kids to wear sunscreen. I could identify with the group It feels like a community of parents that care about their kid	7
Sharing content with family	The articles we’ve been able to share with our loved ones	4
Dislike	Too many posts Not appropriate to be posting something that I consider work on the weekends, especially early on Sunday mornings Stay on topic about sun safety, don’t get into topics of parent advice, co-parenting.	6

### Effects on Parent-Rated Child Sun Protection and Exposure and Individual and Interpersonal Factors

Pre-post changes in all child sun protection outcomes were significant (*P*<.01; see Table 5). The values for Cohen *d* indicated moderate effect-sized increases in child sun protection and decreases in sunburn as well as small to moderate effect-sized decreases in weekday and weekend UV radiation exposure. Parent planning for child sun protection and parent self-efficacy for implementing child sun protection increased significantly (*P*<.05), with small to moderate effect-sized changes. Family norms for sun protection and parent communication and facilitation of child sun protection increased significantly (*P*<.01), with moderate effect-sized effects. Pre-post changes in knowledge, risk, barriers, habits, and peer norms were not significant.

**Table 5:** Changes from baseline to follow-up on primary and secondary outcomes.

Outcome			Pre-intervention, mean (SE)		Post-intervention, mean (SE)		Difference, mean (SE)		*P* value		Cohen *d*^a^
**Sun protection outcomes**											
	Child sun protection^b^	2.60 (0.07)		3.04 (0.08)		0.45 (0.22)		<.001***		0.69	
	Child sun exposure (weekdays)^c^	2.85 (0.12)		2.38 (0.13)		–0.47 (0.18)		.009**		0.33	
	Child sun exposure (weekends)^c^	3.54 (0.16)		2.93 (0.17)		–0.61 (0.23)		.009**		0.34	
	Child sunburn^d^	1.01 (0.10)		0.36 (0.11)		–0.65 (0.15)		<.001***		0.50	
**Individual factors**											
	Child sun safety knowledge^e^	5.25 (0.12)		5.48 (0.12)		0.23 (0.17)		.17		0.14	
	Child skin cancer risk	2.65 (0.11)		2.64 (0.11)		–0.01 (0.02)		.96		0.02	
	Barriers for child sun protection^e^	2.63 (0.07)		2.49 (0.08)		–0.14 (0.11)		.18		0.19	
	Self-efficacy for child sun protection^f^	3.53 (0.09)		3.81 (0.09)		0.28 (0.13)		.03*		0.40	
	Planning for child sun protection^f^	3.67 (0.10)		4.25 (0.10)		0.59 (0.14)		<.001***		0.58	
	Sun protection habits^f^	3.85 (0.10)		3.98 (0.10)		0.13 (0.14)		.36		0.17	
**Interpersonal factors**											
	Family sun protection norms^f^	3.16 (0.06)		3.49 (0.06)		0.34 (.09)		<.001***		0.51	
	Peer sun protection norms^f^	4.24 (0.07)		4.41 (0.07)		0.17 (0.10)		.09		0.20	
	Parent facilitation and communication^f^	3.53 (0.09)		3.87 (0.09)		0.34 (0.13)		.009**		0.51	

^a^For effect sizes, small (*d*=0.2), medium (*d*=0.5), and large (*d*=0.8) suggested by Cohen [42].

^b^Child sun protection behaviors scale range=1-5.

^c^Child sun exposure scale range=1-7.

^d^Child sunburn scale range=0-6.

^e^Knowledge scale range=0-8.

^f^Risk, barriers, efficacy planning, habits, norms, facilitation, and communications scales range=1-5.

*P<.05.

**P<.01.

***P<.001.

## Discussion

There are relatively few parent-focused child sun protection interventions or those specifically targeting parents of children in middle childhood. This pilot study confirmed that the SSF intervention delivered via social media was feasible, acceptable, and satisfying to parents. In terms of feasibility, half of the parents, who were passed to the study team by Qualtrics, enrolled. This enrollment level was lower than our benchmark (73.9%, 68/92) and lower than some other studies using online panels for behavioral intervention trials [43,44]. A potential reason for lower enrollment is that potential participants may not have been interested in joining an intervention study due to the time commitment, as many studies through Qualtrics involve a 1-time survey. A Facebook intervention could also raise concerns about privacy among participants. Another reason is that parents may have less time for additional activities than nonparents, such as joining a research study. However, in terms of retention, the 95% (87/92) posttest survey completion rate was higher than our benchmark and suggested that once recruited, parents were committed to completing the study.

Acceptability, as defined as activity in the Facebook groups, and satisfaction, defined by the treatment evaluation, with the group were good. On average, participants viewed 67.6% (56.8/84) of the posts. A total of 41% (38/92) participants viewed between 89% (75/84) and 100% (84/84) of posts. Although likes and comments were less common, 55.4% (51/92) of parents responded to more than half of the polls. Still, few parents were willing to engage by contributing to the social media feed. Polls may be an effective strategy to engage participants in a Facebook intervention, possibly because they take very little active effort compared to formulating a comment and have a low risk of perceived judgment by other group members. It is difficult to make comparisons with other Facebook studies because engagement has been calculated differently across studies, with many studies characterizing the percentage or average number of likes, comments, and views separately for different types of postings (eg, information, links, videos, pictures, questions, challenges, and polls) [45-47]. At 14.8% (12.43/84), our average for the percentage of comments was slightly higher than a prior study (9.9%) [48]. Satisfaction was high on both quantitative indicators and open-ended feedback. Participants provided the most positive comments about the information provided, along with feeling they were able to communicate more effectively with their children about sun safety. There was very little negative feedback, although several parents noted that they did not see the posts come up on their feed, did not like the non-sun safety posts, or were too busy to participate actively. The Facebook algorithm can de-emphasize content from feeds with low engagement, making additional successful engagement strategies essential to make health behavior intervention effective.

The secondary aim was to evaluate the impact of SSF on parent-rated child sun protection, exposure, and sunburn. Our results were promising, as there were moderate-sized increases in parent-rated child sun protection and declines in sunburn. Consistent with the focus of SSF’s intervention, parents reported increased planning for child sun protection, confidence in their ability to implement child sun protection, family sun protection norms, and communication about and facilitation of their child’s sun protection. However, knowledge, risk, barriers, and habits did not change significantly after intervention. Pre-intervention knowledge scores were relatively high (5.25/8, 64.6% correct), and therefore parents may have demonstrated a ceiling effect in their knowledge gains. Average levels of pre-intervention perceived risk were approximately at the mid-point and did not increase. It is possible that parents recognize the UV radiation risk for their children, but sun safety implementation is challenging. The child’s skin cancer risk is so far in the future that managing the immediate barriers might pose a more immediate challenge for parents. One explanation for the lack of reduction in sun protection barriers and increases in sun safety habits may be that there was only 1 explicit post about how to form sun safe habits and only 1 explicit post on barriers to child sun protection. In our future work, it may be important to add messages on ways of addressing the child’s barriers to sun protection, and methods for forming sun safe habits with the child.

This study had several limitations. First, this was a noncontrolled pilot study. Therefore, we do not know if sun protection and exposure would have changed without intervention. Second, we did not collect child-rated sun protection and exposure, knowledge, or attitudes about sun protection, which may have provided more accurate information about sun protection that occurred when the child was out of the parent’s presence or about changes in child’s knowledge and attitudes. Third, the sample of parents and children was composed primarily of non-Hispanic White mothers and non-Hispanic White children. Although skin cancer is more prevalent among non-Hispanic White populations, it is increasing among Hispanic populations [8]. Future research should focus more on recruiting Hispanic families as well as translating the intervention and survey into Spanish. Fourth, prospective participants were recruited by a third party, Qualtrics, and subsequently contacted and enrolled by study staff. Although Qualtrics recruits a nationally representative panel and is widely used in behavioral research, this approach may have resulted in a biased sample of those who actually enrolled in the study (ie, willing to participate in an intervention study or interested in parenting advice on sun protection). Future studies could directly recruit participants via social media advertisements or other direct methods, but they too may experience similar selection biases related to participant preferences. Fifth, measurement might be improved by using observational methods, such as visual observation, personal UV radiation dosimeters, or the use of spectrophotometers to measure skin color change. The use of more objective methods could address the demand characteristics inherent to a self-report measure when the objective of improving sun safety is known to participants. However, gaining access to outdoor venues with children (eg, school playground) can be challenging, and personal UV radiation dosimeters mainly assess UV radiation exposure and not sun protection behaviors. It can also be challenging and expensive to implement spectrophotometers, which require children to attend in-person assessment sessions. Encouragingly, measures of sun protection and exposure have shown adequate psychometric properties in the past [31,49-52]. In the context of this study, self-reports were more appropriate for assessing the cognitive processes of interest than observations. Finally, this intervention study was conducted in the late summer months when ambient UV levels were declining. Cooler temperatures may reduce concerns about sun safety. Future studies would benefit if they were conducted in spring and in early and midsummer months, to allow for the collection of intervention outcomes when ambient UV radiation is high.

This pilot study of a Facebook parent sun protection intervention suggests that it was feasible and acceptable to a national sample of parents and had a beneficial impact on children’s sun protection and sun exposure and increased family norms, communication about sun protection with the child, and parent’s ability to plan for better sun protection. These changes are encouraging as this is a developmental period when children are taking more responsibility for their own health behaviors, spending more time away from their parents, and starting to build lifelong habits. A future efficacy trial is needed to compare SSF with an active control condition and assess long-term effects.

## Abbreviations

CONSORT: Consolidated Standards of Reporting Trials
EST: Ecological Systems Theory
IBM: Integrative Behavioral Model
SPF: sun protection factor
SSF: Sun Safe Families Facebook

## Data Availability

The data underlying this study cannot be shared publicly to protect the privacy of the individuals that participated in the study. The data will be shared on reasonable request to the corresponding author.
